# Supplementary material for: Complement C3 identified as a unique risk factor for disease severity among young COVID-19 patients in Wuhan, China
Source: Sci Rep. 2021 Apr 12;11:7857. doi: 10.1038/s41598-021-82810-3 (PMC8042103; doi:10.1038/s41598-021-82810-3)
Supplement: Supplementary file 1 — Supplementary Information. [file 41598_2021_82810_MOESM1_ESM.docx]

**Complement C3 identified as a unique Risk Factor for Disease Severity among Young COVID-19 Patients in Wuhan, China - Supplement: Detailed descriptions of the statistical analyses**

Running title: Complement C3 is a unique risk factor for young COVID-19 patients

Weiting Cheng MD ^1^, Roman Hornung PhD ^2^, Kai Xu MD ^3, #^ , Jian Li PhD ^4^

^1^ Oncology Department, Wuhan No.1 Hospital, Wuhan, 430022, China

^2^ Institute of Medical Information Processing, Biometry and Epidemiology, Ludwig-Maximilian-University Munich, Germany

^3^ Department of orthopedics, Tongji hospital, Huazhong University of Science and Technology, Wuhan, 430030, China.

^4^ Institute of Experimental Immunology, University Clinic of Rheinische Friedrich-Wilhelms-University, Bonn, Germany

Weiting Cheng: joycvt@126.com; Roman Hornung: hornung@ibe.med.uni-muenchen.de; Jian Li: jianli@uni-bonn.de

^#^ Corresponding author: Kai Xu, Email: godocoto@163.com; Tel: +86-027-83665418

Tongji Hospital, Huazhong University of Science and Technology

Jiefang Avenue 1095

Wuhan 430030

Province Hubei

China

Words: 3000

number of references: 24

number of tables: 5

number of figures: 2

**Key words** COVID-2019; SARS-CoV-2; young patients; prognosis; severe disease course

**Detailed description of the statistical analysis flow**

A total of 42 demographic characteristics, clinical information, vital signs and laboratory values were analyzed for a possible association with the outcome "severe vs. mild" in univariable and multivariable analyses. Information on all of these 42 variables, denoted as *covariates* in the following, and the outcomes is provided in Table 1 in the main paper.

Data preprocessing

As a first step, all categorical covariates, for which one of the two classes (all categorical covariates were binary) was represented by less than 10 observations in the subgroup of the patients with at most 60 years of age were excluded. The latter subgroup will be referred to as the "*young patients*" in the following. In contrast, the subgroup of patients older than 60 years will be denoted as the "*elderly patients*". For several metric covariates, very small or very high values were not given in the data, but it was only stated that these values are below or above a certain limit, respectively; for example, myohemoglobin values above 1200 were given in the form ">1200". For most covariates, for which this was the case, the fraction of values not provided exactly was small. As the analyses required that fixed numeric values are used, entries that stated that a value was beyond a limit were set equal to the limit; for example, ">1200" was replaced by "1200". As the true values are more extreme, this strategy can be expected to deliver conservative results, as the observed dependencies between the covariates and the outcome will not be dominated by extreme measurements with unknown true values. Subsequently, boxplots of the metric covariates were visually examined for extremely outlying values, which were then deleted and treated as missing values in order to avoid the risk of obtaining distorted results, heavily influenced by outlying values.

There were two pairs of metric covariates with a Pearson correlation *ρ* larger than 0.9: 1) ratio of CRP versus ALB, CRP (*ρ* = 0.97); 2) WBC, ANC (*ρ* = 0.94). Strongly correlating pairs of variables can lead to suboptimal results of analyses that consider multiple covariates together, so-called *multivariable analyses*, because the statistical information in both members of a strongly correlated pair of variables is nearly identical. The latter can lead to high variability in the obtained results. For this reason, in the multivariable analyses CRP and ANC were not considered. However, given the strong correlations within the pairs of variables mentioned above, the conclusions drawn for the ratio of CRP versus ALB / WBC would have been very similar for CRP / ANC, if the latter would have been used instead.

Missing data imputation

Several covariates in the data contained varying percentages of missing values. In the multivariable analyses it was required that all values are present for each patient. Multiple imputation using the popular MICE approach^1^ was performed to deal with this issue. With multiple imputation, the missing values in the data set are repeatedly filled up with plausible values, where, in simplified terms, the latter are, for each patient, educated guesses based on the values of the subset of covariates observed for that patient. This repeated filling up of the missing values leads to several complete versions of the data set that differ with respect to the values used for the missing values. These completed versions of the data set will in the following be denoted as the *imputed data sets*. Depending on the analysis, the imputed data sets are analyzed separately and the results averaged or they are combined and analyzed simultaneously. The purpose of considering several versions of the data set with different values used for the missing values is to decrease the variability of the results caused by the fact that the inserted values are merely guesses of the true values. Moreover, when analyzing the imputed data sets separately it is possible to quantify the uncertainty of the results caused by the missing data. Some covariates featured extremely skew distributions. These were log-transformed during the imputation and re-transformed to the original scale after imputation. A visual inspection confirmed that this proceeding improved the similarity of the distributions of the imputed values to that of the observed values. Twenty imputed data sets were used for each analysis.

Univariable analysis

In order to obtain a first understanding of how the covariates influence the outcome "severe vs. mild" the influences of all covariates on the outcome were analyzed in an univariable fashion using logistic regression. This analysis was performed for all patients taken together and separately for the young and the elderly patients. For each of the three sub-analyses the *p*-values were adjusted for multiple testing using the Benjamini-Hochberg procedure. As the covariates are considered one at a time in an univariable analysis, multiple imputation was not necessary here. Instead, when analyzing the influence of each covariate, the observations with missing values for that covariate were excluded.

Multivariable analysis

Covariate importance values and forms of influences of the covariates

The univariable analysis was used to determine, which of the covariates influence the outcome. In addition, the odds ratios obtained in the logistic regressions give indications of the nature of the influences of the covariates on the outcomes.

However the univariable analysis is not suitable for determining, which of the covariates are most important for predicting the values of the outcomes. A second issue is that the influences of the covariates that can be rendered by the odds ratios from logistic regression are restricted to a specific form, which is why they frequently deliver an incorrect picture of the actual influence patterns. More precisely, the odds ratios describe linear influences of the covariates on the log odds of the outcome. The actual forms of the influences of the (metric) covariates are, however, generally more complex than simple linear influences.

In order to measure the importances of the covariates and their influence patterns, the popular random forest approach^2^ was used. The latter involves a metric for measuring and ranking the covariates with respect to their importance in predicting the outcome, the permutation variable importance measure (VIM). The original version of this measure is suboptimal in the presence of strongly unbalanced outcome classes and an improved measure, the AUC-VIM, was introduced^3^, which does not suffer from this drawback. The AUC-VIM was used in this study. The variable importance measure is also affected by a selection bias in the original random forest algorithm, which can lead to an underestimation of the importance of categorical covariates. Therefore, in this study conditional inference trees^4^ were used as base learners in the random forests for measuring the variable importance, because this kind of trees is not affected by selection bias. The influence patterns of the covariates on the outcomes was measured using partial dependence plots^5^ obtained from random forests. Partial dependence plots show the predictions of a classifier (e.g., of random forests) as a function of an individual covariate of interest after marginalizing out the influences of the remaining covariates. Given the fact that random forests are able to capture complex dependency patterns between the outcome and the covariates, partial dependence plots obtained using random forests are particularly useful for learning about the actual influences of the covariates.

Random forest was applied to each of the 20 imputed data sets from the multiple imputation, resulting in 20 AUC-VIM values and partial dependence plots for each of the covariates. In order to obtain a single AUC-VIM value for each covariate, the median of the 20 AUC-VIM values associated with that covariate was taken. Taking the median seemed more appropriate than taking the mean, because the AUC-VIM values obtained for some imputed data sets were outlying. The 20 partial dependence plots obtained for each covariate were averaged on the log odds scale of the probability predictions for the outcome categories and subsequently re-transformed to the probability scale.

AUC-VIM values and partial dependence plots were obtained for all patients irrespective of age and separately for young and elderly patients in order to identify risk factors specific to young patients. In the latter analysis, the imputation was performed separately for young and elderly patients in order to account for the possibility that the dependency structures between the variables might differ between young and elderly patients.

Prognostic model building

The goals of the prognostic model building were to gain knowledge on which combinations of covariates are most suited to predict a severe outcome and to obtain models that can be used in practice for predicting the risks of patients to experience a severe outcome.

Prognostic models were obtained both, specifically for young patients and for all patients irrespective of age. In both cases logistic regression models as well as random forests were considered.

Covariate selection in the logistic regression modeling was performed using a forward selection procedure applicable to multiply imputed data^6^. As performance criterion the Akaike information criterion (AIC) was used. Note that stepwise regression approaches, to which forward selection belongs, receive criticism, because they do not take the uncertainty in the model selection process into account, causing the coefficient estimates to be biased^7^. This is a problem in the presence of many covariates in combination with a comparably low sample size, in which case penalization approaches that perform covariate selection and parameter estimation at the same time, such as LASSO^8^, should be applied. However, the coefficient estimates obtained from penalization approaches are biased as well, in particular also in the case of smaller numbers of covariates compared to the sample size. In the present study, the number of observations is large in comparison to the number of covariates. For this reason, the variability associated with selecting the best model using forward selection can be expected to be low. Therefore, the selected models will be close to the truly best models, which is why it is less problematic that the variability in the model selection is not taken into account. Apart from the fact that the parameter estimates obtained with penalization approaches are biased independent from the number of covariates considered, there is another disadvantage of such approaches in the context of the present study: all covariates, that is, also those with large proportions of missing values are included throughout the estimation procedure. With forward selection in contrast, the estimation is more stable, because it is likely that covariates with large proportions of missing values that do not contribute much to explaining the outcome are not included in the stepwise procedure.

The stepwise regression approach for multiply imputed data that was used in this study consists of performing stepwise regression using AIC (or BIC; see further down) as usual, while replacing the unknown log-likelihood of the full data (that includes the unknown values in the missing entries) by the arithmetic mean of the log-likelihoods of the imputed data sets multiplied by the proportion of observed values in the data^6^. The latter is applied easily in R using the function 'step()' for stepwise regression from the 'stats' package (version 3.6.2): To perform backward selection using multiply imputed data, first, using the 'glm()' function, a standard logistic regression model including all covariates has to be fit to the data frame of all imputed data sets stacked together; second, 'step()' has to be applied to the result and the input parameter 'k' set to 2*M/f for selection using the AIC (to log(n)*M/f for selection using the BIC). Here, M denotes the number of imputed data sets, f the proportion of observed values and n the number of observations in the original data set.

For both logistic regression models (one specifically for young patients and one for all patients irrespective of age), as a sensitivity analysis^9^, backward selection was applied in addition to forward selection and the Bayesian information criterion (BIC) used instead of the AIC in order to investigate the robustness of the selected models with respect to the selection method used.

Random forests including all covariates were constructed: 1) a separate random forest with 1000 trees and 'mtry' set to the square root of the number of covariates was grown using each of the 20 imputed data sets; 2) the trees in the 20 random forests constructed in 1) were combined to form a single forest. Given that random forests outperform logistic regression for most data sets^1^^0^ and that the random forests were constructed using all covariates, it was expected that the latter would be associated with a better prediction performance than the logistic regression models. The random forests were mainly included to make it possible to compare the prediction performance of the logistic regression models against a gold standard, because it was expected that the prediction performance obtained through the random forests would be close to the best achievable prediction performance. However, in practice it might be difficult to apply the random forests, because they use the values of all covariates and are only applicable through the use of computers.

The prediction performance of the models was estimated using 20 times repeated stratified K-fold cross-validation, repeating the whole model selection process on each training set in each cross-validation iteration, excluding the corresponding test set. Wahl et al.^1^^1^ found that using cross-validation after having performed multiple imputation on the whole data sets leads to optimistically biased performance estimates, while those obtained when performing the imputation within cross-validation separately on the training and test sets are largely unbiased. Therefore, the latter strategy was followed in this study. Note that Wahl et al. also found that sampling the training sets using bootstrapping and using the out-of-bag observations to be the most reliable validation procedure. However, this validation procedure was not considered in this study. The reason for this is that the AIC and BIC were used to select the covariates in the logistic regression models: Janitza et al.^1^^2^ found that information criteria such as the AIC and BIC are optimistically biased on bootstrap samples, which would have led to the selection of overly complex models.

The values 3, 4, and 5 were considered for K in the stratified K-fold cross-validation. The reason for considering different values of K was to study the sensitivity of the performance estimates with respect to the choice of this value. Choosing a small value for K is associated with small training sets, for which the obtained models may not feature a prediction performance that is comparable to that of the model obtained on the whole data set. On the other hand, choosing a large value of K is associated with small test data sets. In this situation, the imputation may lead to suboptimal results on the test data, because the parameters of the multiple imputation procedure are estimated with high variance, if the number of observations is small.

For measuring the performance in the cross-validation both the AUC and the Brier score were used. While the AUC has the advantage that its values are well interpretable, it has the disadvantage that it only measures, how well the prognostic model performs with respect to ranking different patients with respect to their risks. However, it does not measure the quality of the probability predictions of the model. A prognostic model can be associated with a high AUC value even though it delivers strongly biased probability predictions that are not useable in clinical practice. The Brier score by contrast is a proper measure for the quality of probability predictions.

**Subgroup analysis of the influence of complement C3 in young patients**

In order to analyze whether complement C3 has a different influence in specific subgroups defined by age, gender, and comorbidities, again logistic regression and random forest analysis were performed.

Because there were no missing values in the variables age and gender and in the variables that provide information on the presence of comorbidities, which were hypertension, diabetes, coronary heart disease, and thyroid related diseases, in this analysis no imputation, but instead a complete case analysis, was performed. That is, all observations for which complement C3 was not given were excluded. As the case numbers with the different types of comorbidities for which complement C3 was given were small, the variables associated with the different comorbidities were not analyzed separately, but it was only considered whether at least one of the comorbidities was present or not.

In the first part of the analysis, for each of the variables age, gender, and comorbidities (yes vs. no) separately, logistic regression was applied to test for an interaction effect between the influence of complement C3 and the corresponding variable. In the latter logistic regression models, apart from the interaction term between complement C3 and the corresponding variable, both of the latter were included as main effects. Subsequently, the three *p*-values associated with the interaction terms for each of the three tested variables were adjusted for multiple testing using the Benjamini-Hochberg procedure. None of the three interaction terms was significant.

In the second part of the analysis, partial dependence plots showing the bivariate influences of complement C3 and each of the three variables age, gender, and comorbidities (yes vs. no) were calculated using random forest (number of trees: 2000). The covariates considered in the random forest were complement C3, age, gender, and comorbidities (yes vs. no). This analysis again suggested that the influence of complement C3 does not notably depend on age, gender or the presence or absence of comorbidities.

**Model stability analysis**

Given that the number of observations available for the variable selection in the model specifically obtained for young patients is limited and that the AIC tends to select a large number of covariates, the model selected using backward selection might be overly large. To obtain indications on whether the model selected for young patients using backward selection might be less stable than the model selected using forward selection, as a sensitivity analysis both models were re-fit on each of the 20 imputed data sets and *p*-values were calculated using Rubin's rules^1^^3^. Here, indeed five of the nine covariates selected by backward selection were associated with a *p*-value larger than 0.05, while this was the case only for one of the six covariates selected by forward selection. More precisely, from the model selected by backward selected the influences of hypertension, thyroid related disease and BUN were not significant and that of gender and SII only weakly significant (0.05 < *p* < 0.10). From the model selected by forward selection, the influence of UA was not significant. Note that the obtained *p*-values may still be optimistic, because, when performing inference for a fixed model selected using a data-driven procedure such as backward selection, the uncertainty in the model selection is not taken into account.

**References:**

1. van Buuren, S., Groothuis-Oudshoorn, K. mice: Multivariate Imputation by Chained Equations in R. *Journal of Statistical Software* 2011;45(3):1-67. doi: 10.18637/jss.v045.i03

2. Breiman, L. Random Forests. *Machine Learning* 2001;45:5-32. doi: 10.1023/A:1010933404324

3. Janitza, S., Strobl, C., Boulesteix, A.-L. An AUC-based permutation variable importance measure for random forests. *BMC Bioinformatics* 2013;14(1):119.

4. Hothorn, T., Hornik, K., Zeileis, A. Unbiased Recursive Partitioning: A Conditional Inference Framework. *Journal of Computational and Graphical Statistics* 2006;15(3):651-74.

5. Friedman, J. Greedy Function Approximation: A Gradient Boosting Machine. *The Annals of Statistics* 2001;29:1189-232.

6. Wood, A. M., White, I. R., Royston, P. How should variable selection be performed with multiply imputed data? *Statistics in Medicine* 2008;27:3227-46. doi: 10.1002/sim.3177

7. Miller, A. J. Selection of Subsets of Regression Variables. *Journal of the Royal Statistical Society Series A* 1984;147(3):389-425. doi: 10.2307/2981576

8. Tibshirani, R. Regression Shrinkage and Selection via the Lasso. *Journal of the Royal Statistical Society Series B* 1996;58(1):267-88.

9. Thabane, L., Mbuagbaw, L., Zhang, S., et al. A tutorial on sensitivity analyses in clinical trials: the what, why, when and how. *BMC Medical Research Methodology* 2013;13:92. doi: 10.1186/1471-2288-13-92

10. Couronne, R., Probst, P., Boulesteix, A. L. Random forest versus logistic regression: a large-scale benchmark experiment. *BMC Bioinformatics* 2018;19(1):270. doi: 10.1186/s12859-018-2264-5

11. Wahl, S., Boulesteix, A. L., Zierer, A., et al. Assessment of predictive performance in incomplete data by combining internal validation and multiple imputation. *BMC Med Res Methodol* 2016;16(1):144. doi: 10.1186/s12874-016-0239-7

12. Janitza, S., Binder, H., Boulesteix, A. L. Pitfalls of hypothesis tests and model selection on bootstrap samples: Causes and consequences in biometrical applications. *Biom J* 2016;58(3):447-73. doi: 10.1002/bimj.201400246

13. Rubin, D.B. Multiple Imputation for Nonresponse in Surveys: New York: John Wiley and Sons 1987.
