# Supplementary material for: Complement C3 identified as a unique risk factor for disease severity among young COVID-19 patients in Wuhan, China
Source: Sci Rep. 2021 Apr 12;11:7857. doi: 10.1038/s41598-021-82810-3 (PMC8042103; doi:10.1038/s41598-021-82810-3)
Supplement: Supplementary file 2 — Supplementary Figures. [file 41598_2021_82810_MOESM2_ESM.docx]

**Complement C3 identified as a unique Risk Factor for Disease Severity among Young COVID-19 Patients in Wuhan, China - Supplementary Figures**

Running title: Complement C3 is a unique risk factor for young COVID-19 patients

Weiting Cheng MD ^1^, Roman Hornung PhD ^2^, Kai Xu MD ^3, #^ , Jian Li PhD ^4^

^1^ Oncology Department, Wuhan No.1 Hospital, Wuhan, 430022, China

^2^ Institute of Medical Information Processing, Biometry and Epidemiology, Ludwig-Maximilian-University Munich, Germany

^3^ Department of orthopedics, Tongji hospital, Huazhong University of Science and Technology, Wuhan, 430030, China.

^4^ Institute of Experimental Immunology, University Clinic of Rheinische Friedrich-Wilhelms-University, Bonn, Germany

Weiting Cheng: joycvt@126.com; Roman Hornung: hornung@ibe.med.uni-muenchen.de; Jian Li: jianli@uni-bonn.de

^#^ Corresponding author: Kai Xu, Email: godocoto@163.com; Tel: +86-027-83665418

Tongji Hospital, Huazhong University of Science and Technology

Jiefang Avenue 1095

Wuhan 430030

Province Hubei

China

Words: 3000

number of references: 24

number of tables: 5

number of figures: 2

**Key words** COVID-2019; SARS-CoV-2; young patients; prognosis; severe disease course

**Supplement Figures**

Supplementary Figure 1: Partial dependence plots (PDPs) for young and elderly patients calculated using random forests – metric covariates (I). In simplified terms, a PDP shows the influence of a covariate on the outcome after adjusting for the influences of the other covariates. The PDPs are ordered by decreasing importance with respect to the covariate importance values of the young patients in Figure 1. The light lines show the 20 individual PDPs calculated using the imputed data sets from the multiple imputation. The bold lines show averages over the 20 individual PDPs.

Supplementary Figure 2: Partial dependence plots (PDPs) for young and elderly patients calculated using random forests – metric covariates (II). In simplified terms, a PDP shows the influence of a covariate on the outcome after adjusting for the influences of the other covariates. The PDPs are ordered by decreasing importance with respect to the covariate importance values of the young patients in Figure 1. The light lines show the 20 individual PDPs calculated using the imputed data sets from the multiple imputation. The bold lines show averages over the 20 individual PDPs.

Supplementary Figure 3: Partial dependence plots (PDPs) for young and elderly patients calculated using random forests – metric covariates (III). In simplified terms, a PDP shows the influence of a covariate on the outcome after adjusting for the influences of the other covariates. The PDPs are ordered by decreasing importance with respect to the covariate importance values of the young patients in Figure 1. The light lines show the 20 individual PDPs calculated using the imputed data sets from the multiple imputation. The bold lines show averages over the 20 individual PDPs.

Supplementary Figure 4: Partial dependence plots (PDPs) for young and elderly patients calculated using random forests – categorical covariates. In simplified terms, a PDP shows the influence of a covariate on the outcome after adjusting for the influences of the other covariates. The PDPs are ordered by decreasing importance with respect to the covariate importance values of the young patients in Figure 1. The bars show averages over the 20 individual PDPs calculated using the imputed data sets. The grey lines show the 20 individual PDPs.

Supplementary Figure 5: AUC variable importance with respect to predicting the outcome "severe vs. mild" for all patients calculated using random forests. The larger the importance value of a covariate is, the greater the improvement of prediction performance by including this covariate in prognosis tends to be. The bars show the medians of the 20 importance values calculated using the 20 imputed data sets from the multiple imputation. The error bars illustrate the variabilities of the importance values: The lower / upper ends show the first / third quartiles of the 20 importance values, that is, 25% percent of the importance values lie below / above these values.

Supplementary Figure 6: Partial dependence plots (PDPs) for all patients calculated using random forests – metric covariates (I). In simplified terms, a PDP shows the influence of a covariate on the outcome after adjusting for the influences of the other covariates. The PDPs are ordered by decreasing importance with respect to the covariate importance values in Supplementary Figure 5. The grey lines show the 20 individual PDPs calculated using the imputed data sets from the multiple imputation. The bold lines show averages over the 20 individual PDPs.

Supplementary Figure 7: Partial dependence plots (PDPs) for all patients calculated using random forests – metric covariates (II). In simplified terms, a PDP shows the influence of a covariate on the outcome after adjusting for the influences of the other covariates. The PDPs are ordered by decreasing importance with respect to the covariate importance values in Supplementary Figure 5. The grey lines show the 20 individual PDPs calculated using the imputed data sets from the multiple imputation. The bold lines show averages over the 20 individual PDPs.

Supplementary Figure 8: Partial dependence plots (PDPs) for all patients calculated using random forests – metric covariates (III). In simplified terms, a PDP shows the influence of a covariate on the outcome after adjusting for the influences of the other covariates. The PDPs are ordered by decreasing importance with respect to the covariate importance values in Supplementary Figure 5. The grey lines show the 20 individual PDPs calculated using the imputed data sets from the multiple imputation. The bold lines show averages over the 20 individual PDPs.

Supplementary Figure 9: Partial dependence plots (PDPs) for all patients calculated using random forests – metric covariates (IV). In simplified terms, a PDP shows the influence of a covariate on the outcome after adjusting for the influences of the other covariates. The PDPs are ordered by decreasing importance with respect to the covariate importance values in Supplementary Figure 5. The grey lines show the 20 individual PDPs calculated using the imputed data sets from the multiple imputation. The bold lines show averages over the 20 individual PDPs.

Supplementary Figure 10: Partial dependence plots (PDPs) for all patients calculated using random forests – categorical covariates. In simplified terms, a PDP shows the influence of a covariate on the outcome after adjusting for the influences of the other covariates. The PDPs are ordered by decreasing importance with respect to the covariate importance values in Supplementary Figure 5. The bars show averages over the 20 individual PDPs calculated using the imputed data sets. The grey lines show the 20 individual PDPs.


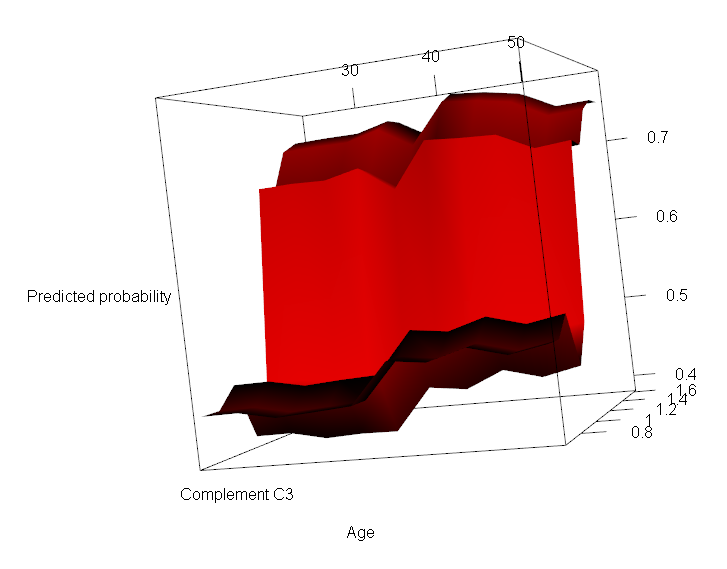


Supplementary Figure 11: Partial dependence plot (PDP) of the bivariate influence of complement C3 and age in young patients. The z axis "Predicted probability" shows the values of the PDP, that is, the predicted probability of a severe outcome (after marginalization). In simplified terms, a bivariate PDP shows the simultaneous influence of two covariates on the outcome after adjusting for the influences of the remaining covariates.

Supplementary Figure 12: Partial dependence plot (PDP) of the bivariate influence of complement C3 and gender in young patients. In simplified terms, a bivariate PDP shows the simultaneous influence of two covariates on the outcome after adjusting for the influences of the remaining covariates.

Supplementary Figure 13: Partial dependence plot (PDP) of the bivariate influence of complement C3 and comorbidities in young patients. In simplified terms, a bivariate PDP shows the simultaneous influence of two covariates on the outcome after adjusting for the influences of the remaining covariates.
