# Supplementary material for: Complement C3 identified as a unique risk factor for disease severity among young COVID-19 patients in Wuhan, China
Source: Sci Rep. 2021 Apr 12;11:7857. doi: 10.1038/s41598-021-82810-3 (PMC8042103; doi:10.1038/s41598-021-82810-3)
Supplement: Supplementary file 3 — Supplementary Tables. [file 41598_2021_82810_MOESM3_ESM.docx]

**Complement C3 identified as a unique Risk Factor for Disease Severity among Young COVID-19 Patients in Wuhan, China - Supplementary Tables**

Running title: Complement C3 is a unique risk factor for young COVID-19 patients

Weiting Cheng MD ^1^, Roman Hornung PhD ^2^, Kai Xu MD ^3, #^ , Jian Li PhD ^4^

^1^ Oncology Department, Wuhan No.1 Hospital, Wuhan, 430022, China

^2^ Institute of Medical Information Processing, Biometry and Epidemiology, Ludwig-Maximilian-University Munich, Germany

^3^ Department of orthopedics, Tongji hospital, Huazhong University of Science and Technology, Wuhan, 430030, China.

^4^ Institute of Experimental Immunology, University Clinic of Rheinische Friedrich-Wilhelms-University, Bonn, Germany

Weiting Cheng: joycvt@126.com; Roman Hornung: hornung@ibe.med.uni-muenchen.de; Jian Li: jianli@uni-bonn.de

^#^ Corresponding author: Kai Xu, Email: godocoto@163.com; Tel: +86-027-83665418

Tongji Hospital, Huazhong University of Science and Technology

Jiefang Avenue 1095

Wuhan 430030

Province Hubei

China

Words: 3000

number of references: 24

number of tables: 5

number of figures: 2

**Key words** COVID-2019; SARS-CoV-2; young patients; prognosis; severe disease course

**Supplement Tables**

|  | All patients | | Young patients (<= 60 years) | | Elderly patients (> 60 years) | |
| --- | --- | --- | --- | --- | --- | --- |
| variable | Odds ratio [CI] | adj. p | Odds ratio [CI] | adj. p | Odds ratio [CI] | adj. p |
| Accu Troponin [μg/L] | 10.1662 [1.0545, 203.6688] | 0.1407 | 6.2231 [0.0224, 7609.7911] | 0.7825 | 7.7080 [0.6814, 231.7369] | 0.2701 |
| ALB [g/L] | 0.9320 [0.9109, 0.9530] | <0.0001 | 0.9501 [0.9170, 0.9831] | 0.0317 | 0.9211 [0.8900, 0.9521] | <0.0001 |
| ALT [U/L] | 1.0008 [0.9976, 1.0041] | 0.6612 | 1.0009 [0.9969, 1.0051] | 0.7994 | 1.0012 [0.9959, 1.0068] | 0.8090 |
| ANC | 1.1055 [1.0549, 1.1611] | 0.0002 | 1.0794 [0.9937, 1.1773] | 0.2062 | 1.1032 [1.0408, 1.1741] | 0.0062 |
| AST [U/L] | 1.0100 [1.0048, 1.0158] | 0.0011 | 1.0062 [1.0005, 1.0130] | 0.1762 | 1.0153 [1.0066, 1.0251] | 0.0059 |
| Blood glucose [mmol/L] | 1.0229 [0.9598, 1.0930] | 0.5440 | 1.0347 [0.9135, 1.1793] | 0.7994 | 0.9948 [0.9204, 1.0778] | 0.9523 |
| Blood platelet [10^9/L] | 0.9990 [0.9978, 1.0002] | 0.1695 | 0.9990 [0.9972, 1.0008] | 0.4904 | 0.9989 [0.9974, 1.0004] | 0.2713 |
| BUN [mmol/L] | 1.1116 [1.0658, 1.1661] | <0.0001 | 1.0461 [0.9923, 1.1270] | 0.3516 | 1.1324 [1.0730, 1.2051] | 0.0002 |
| CK [U/L] | 1.0023 [1.0011, 1.0036] | 0.0011 | 1.0021 [1.0005, 1.0042] | 0.0926 | 1.0023 [1.0007, 1.0041] | 0.0196 |
| CK-MB [U/L] | 1.0072 [0.9995, 1.0171] | 0.1641 | 1.0020 [0.9916, 1.0134] | 0.7994 | 1.0149 [1.0012, 1.0353] | 0.1431 |
| Complement C3 [g/L] | 2.0055 [0.6022, 6.9684] | 0.3699 | 15.5808 [2.4111, 122.2841] | 0.0392 | 0.3323 [0.0478, 1.9321] | 0.3700 |
| Complement C4 [g/L] | 2.3254 [0.4307, 18.8982] | 0.4435 | 15.9603 [0.7470, 791.5843] | 0.3270 | 0.8382 [0.0820, 9.5655] | 0.9523 |
| Coronary heart disease | 1.2967 [0.8962, 1.8869] | 0.2494 | 1.2342 [0.4920, 3.1404] | 0.7994 | 1.1339 [0.7474, 1.7321] | 0.7364 |
| Cr [μmol/L] | 1.0076 [1.0035, 1.0122] | 0.0017 | 1.0046 [0.9979, 1.0121] | 0.3963 | 1.0083 [1.0030, 1.0143] | 0.0114 |
| CRP | 1.0119 [1.0079, 1.0161] | <0.0001 | 1.0122 [1.0043, 1.0209] | 0.0317 | 1.0110 [1.0064, 1.0161] | 0.0001 |
| D-Dimer [mg/L] | 1.0808 [1.0386, 1.1358] | 0.0017 | 1.0580 [0.9368, 1.2306] | 0.6455 | 1.0770 [1.0319, 1.1374] | 0.0081 |
| Diabetes | 1.1572 [0.8585, 1.5629] | 0.4341 | 1.1607 [0.6560, 2.0575] | 0.7994 | 1.0125 [0.7047, 1.4590] | 0.9563 |
| Erythrocyte sedimentation rate | 1.0076 [0.9996, 1.0159] | 0.1248 | 1.0124 [0.9997, 1.0263] | 0.1980 | 1.0023 [0.9914, 1.0137] | 0.8231 |
| Gender | 0.8927 [0.7271, 1.0957] | 0.3797 | 1.0576 [0.7942, 1.4088] | 0.7994 | 0.7590 [0.5637, 1.0205] | 0.1335 |
| Ground glass opacity | 0.9978 [0.7580, 1.3135] | 0.9874 | 0.8302 [0.5642, 1.2164] | 0.5831 | 1.2130 [0.8147, 1.8152] | 0.5035 |
| Hemoglobin [g/L] | 0.9942 [0.9883, 1.0000] | 0.0986 | 0.9985 [0.9901, 1.0069] | 0.7994 | 0.9924 [0.9838, 1.0008] | 0.1431 |
| Hypertension | 1.1917 [0.9533, 1.4909] | 0.1884 | 0.8871 [0.5885, 1.3318] | 0.7980 | 1.1696 [0.8699, 1.5738] | 0.4557 |
| IgA [g/L] | 1.1442 [0.8988, 1.5032] | 0.3926 | 1.1574 [0.8152, 1.7333] | 0.6896 | 1.1302 [0.8092, 1.6836] | 0.6793 |
| IgG [g/L] | 1.1140 [1.0229, 1.2355] | 0.0573 | 1.0831 [0.9764, 1.2356] | 0.3937 | 1.1633 [1.0148, 1.3991] | 0.1329 |
| IgM [mg/dl] | 1.0048 [0.6451, 1.6096] | 0.9874 | 0.9948 [0.5739, 1.7924] | 0.9849 | 1.0217 [0.4630, 2.4472] | 0.9563 |
| LDH [U/L] | 1.0027 [1.0018, 1.0038] | <0.0001 | 1.0026 [1.0011, 1.0043] | 0.0317 | 1.0027 [1.0015, 1.0040] | 0.0002 |
| LYM [10^9/L] | 0.5842 [0.4926, 0.6908] | <0.0001 | 0.5947 [0.4664, 0.7536] | 0.0009 | 0.6016 [0.4698, 0.7662] | 0.0003 |
| MONO [10^9/L] | 0.8312 [0.5582, 1.2341] | 0.4435 | 0.6439 [0.3400, 1.1952] | 0.3834 | 0.9108 [0.5397, 1.5395] | 0.8503 |
| Multiple patchy shadows | 1.1094 [0.8383, 1.4692] | 0.5440 | 1.1360 [0.7859, 1.6470] | 0.7570 | 1.0276 [0.6637, 1.5872] | 0.9523 |
| Myohemoglobin [ng/mL] | 1.0054 [1.0027, 1.0091] | 0.0024 | 1.0022 [0.9918, 1.0134] | 0.7994 | 1.0055 [1.0025, 1.0096] | 0.0081 |
| PLR | 1.0015 [1.0006, 1.0024] | 0.0040 | 1.0008 [0.9996, 1.0022] | 0.3963 | 1.0017 [1.0005, 1.0031] | 0.0196 |
| Procalcitonin [ug/L] | 1.4658 [1.0756, 2.2227] | 0.0745 | 0.4527 [0.0851, 1.2045] | 0.4262 | 1.8933 [1.1962, 3.8790] | 0.0663 |
| Pulmonary consolidation | 1.0721 [0.6151, 1.8756] | 0.8469 | 0.9426 [0.4229, 2.0698] | 0.9279 | 1.2077 [0.5495, 2.7363] | 0.8090 |
| Ratio of CRP versus ALB | 1.3836 [1.2341, 1.5667] | <0.0001 | 1.3994 [1.0999, 1.8321] | 0.0430 | 1.3393 [1.1759, 1.5467] | 0.0002 |
| Ratio of neutrophile versus lymphocyte | 1.1061 [1.0687, 1.1485] | <0.0001 | 1.0835 [1.0256, 1.1539] | 0.0416 | 1.1105 [1.0629, 1.1666] | 0.0001 |
| RBC [10^12/L] | 0.7775 [0.6493, 0.9287] | 0.0140 | 0.9856 [0.7644, 1.2707] | 0.9337 | 0.6592 [0.5018, 0.8600] | 0.0081 |
| SAA [mg/L] | 1.0065 [1.0032, 1.0100] | 0.0006 | 1.0066 [1.0023, 1.0112] | 0.0317 | 1.0059 [1.0008, 1.0114] | 0.0663 |
| SII | 1.0003 [1.0002, 1.0004] | <0.0001 | 1.0003 [1.0001, 1.0006] | 0.0430 | 1.0003 [1.0001, 1.0004] | 0.0063 |
| Thyroid related diseases | 0.5614 [0.2647, 1.1413] | 0.1854 | 0.3862 [0.1238, 1.0206] | 0.2062 | 0.9357 [0.3078, 2.9334] | 0.9523 |
| UA [μmol/L] | 0.9996 [0.9986, 1.0006] | 0.5440 | 0.9985 [0.9970, 1.0000] | 0.1762 | 1.0006 [0.9992, 1.0020] | 0.6046 |
| WBC [10^9/L] | 1.0425 [1.0012, 1.0864] | 0.0932 | 0.9914 [0.9225, 1.0650] | 0.8775 | 1.0562 [1.0038, 1.1141] | 0.0844 |

Supplementary Table 1: Univariable logistic regression for the outcome "severe vs. mild". The p-values were adjusted for multiple testing separately for the analysis of all patients, young patients, and elderly patients. The confidence intervals were not adjusted for multiple testing, because these serve the mere descriptive purpose of illustrating the uncertainty in the estimates of the true odds ratios.

|  | Regression coefficient | Odds ratio |
| --- | --- | --- |
| Intercept | -1.595926 | - |
| Complement C3 [g/L] | 1.771946 | 5.8823 |
| Gender: female | 0.375952 | 1.4564 |
| Hypertension | -0.458988 | 0.6319 |
| SAA [mg/L] | 0.005695 | 1.0057 |
| BUN [mmol/L] | 0.050977 | 1.0523 |
| LYM [10^9/L] | -0.478214 | 0.6199 |
| PLR | -0.003914 | 0.9961 |
| SII | 0.000399 | 1.0004 |
| Thyroid related diseases | -0.963877 | 0.3814 |

Supplementary Table 2: Multivariable logistic regression models for the outcome "severe vs. mild" in young patients selected using the AIC criterion and backward selection.

|  | Regression coefficient | Odds ratio |
| --- | --- | --- |
| Intercept | -2.05622 | - |
| Complement C3 [g/L] | 1.414744 | 4.1154 |
| SAA [mg/L] | 0.005878 | 1.0059 |

Supplementary Table 3: Multivariable logistic regression models for the outcome "severe vs. mild" in young patients selected using the BIC criterion and backward/forward selection.

|  | Regression coefficient | Odds ratio |
| --- | --- | --- |
| Intercept | 0.366505 | - |
| Complement C3 [g/L] | 0.435939 | 1.5464 |
| IgA [g/L] | -0.137703 | 0.8714 |
| IgG [g/L] | 0.03402 | 1.0346 |
| ALB [g/L] | -0.03952 | 0.9613 |
| SAA [mg/L] | 0.005803 | 1.0058 |
| BUN [mmol/L] | 0.068764 | 1.0712 |
| LDH [U/L] | 0.001673 | 1.0017 |
| LYM [10^9/L] | -0.287398 | 0.7502 |
| PLR | -0.002025 | 0.9980 |
| Blood platelet | 0.001629 | 1.0016 |

Supplementary Table 4: Multivariable logistic regression models for the outcome "severe vs. mild" in all patients irrespective of age selected using the AIC criterion and backward selection.

|  | Regression coefficient | Odds ratio |
| --- | --- | --- |
| Intercept | 0.48423 | - |
| ALB [g/L] | -0.040039 | 0.9608 |
| SAA [mg/L] | 0.005673 | 1.0057 |
| BUN [mmol/L] | 0.065018 | 1.0672 |
| LDH [U/L] | 0.001581 | 1.0016 |

Supplementary Table 5: Multivariable logistic regression models for the outcome "severe vs. mild" in all patients irrespective of age selected using the BIC criterion and backward/forward selection.

|  | Young patients | | | | All patients | | | |
| --- | --- | --- | --- | --- | --- | --- | --- | --- |
|  | AIC | | BIC | | AIC | | BIC | |
|  | FW | BW | FW | BW | FW | BW | FW | BW |
| SAA | 1 | 1 | 1 | 1 | 1 | 1 | 1 | 1 |
| Complement C3 | 1 | 1 | 1 | 1 | 1 | 1 | 0 | 0 |
| PLR | 1 | 1 | 0 | 0 | 1 | 1 | 0 | 0 |
| BUN | 0 | 1 | 0 | 0 | 1 | 1 | 1 | 1 |
| LDH | 0 | 0 | 0 | 0 | 1 | 1 | 1 | 1 |
| ALB | 0 | 0 | 0 | 0 | 1 | 1 | 1 | 1 |
| LYM | 1 | 1 | 0 | 0 | 0 | 1 | 0 | 0 |
| SII | 1 | 1 | 0 | 0 | 0 | 0 | 0 | 0 |
| UA | 1 | 0 | 0 | 0 | 0 | 0 | 0 | 0 |
| Immunoglobulin IgA | 0 | 0 | 0 | 0 | 1 | 1 | 0 | 0 |
| Immunoglobulin IgG | 0 | 0 | 0 | 0 | 1 | 1 | 0 | 0 |
| Gender | 0 | 1 | 0 | 0 | 0 | 0 | 0 | 0 |
| Hypertension | 0 | 1 | 0 | 0 | 0 | 0 | 0 | 0 |
| Thyroid related diseases | 0 | 1 | 0 | 0 | 0 | 0 | 0 | 0 |
| Blood platelet | 0 | 0 | 0 | 0 | 0 | 1 | 0 | 0 |

Supplementary Table 6: Overview on the covariates selected in the different multivariable logistic regression models. Entries of "1" mean that the covariates were selected in the respective models and entries of "0" mean that they were not selected. FW=forward selection, BW=backward selection
